# With No Attention Specifically Directed to It, Rhythmic Sound Does Not Automatically Facilitate Visual Task Performance

**DOI:** 10.3389/fpsyg.2022.894366

**Published:** 2022-06-10

**Authors:** Jorg De Winne, Paul Devos, Marc Leman, Dick Botteldooren

**Affiliations:** ^1^Department of Information Technology, WAVES, Ghent University, Ghent, Belgium; ^2^Department of Art, Music and Theater Studies, Institute for Psychoacoustics and Electronic Music (IPEM), Ghent University, Ghent, Belgium

**Keywords:** audiovisual binding, visual attention, auditory support, rhythmic support, working memory, attentional resources

## Abstract

In a century where humans and machines—powered by artificial intelligence or not—increasingly work together, it is of interest to understand human processing of multi-sensory stimuli in relation to attention and working memory. This paper explores whether and when supporting visual information with rhythmic auditory stimuli can optimize multi-sensory information processing. In turn, this can make the interaction between humans or between machines and humans more engaging, rewarding and activating. For this purpose a novel working memory paradigm was developed where participants are presented with a series of five target digits randomly interchanged with five distractor digits. Their goal is to remember the target digits and recall them orally. Depending on the condition support is provided by audio and/or rhythm. It is expected that the sound will lead to a better performance. It is also expected that this effect of sound is different in case of rhythmic and non-rhythmic sound. Last but not least, some variability is expected across participants. To make correct conclusions, the data of the experiment was statistically analyzed in a classic way, but also predictive models were developed in order to predict outcomes based on a range of input variables related to the experiment and the participant. The effect of auditory support could be confirmed, but no difference was observed between rhythmic and non-rhythmic sounds. Overall performance was indeed affected by individual differences, such as visual dominance or perceived task difficulty. Surprisingly a music education did not significantly affect the performance and even tended toward a negative effect. To better understand the underlying processes of attention, also brain activation data, e.g., by means of electroencephalography (EEG), should be recorded. This approach can be subject to a future work.

## 1. Introduction

Optimizing multi-sensory information processing is, according to different studies and due to many reasons, a challenging task. Consider the so-called “pip and pop” effect. Performance tasks based on the attention to visual stimuli were shown to be affected by simultaneous auditory stimuli, typically consisting of short and simple sounds, called auditory “pips.” When synchronized with the visual stimuli, these sounds drastically decrease the participant's time needed to find the visual changes. The synchronous “pip” makes the visual stimulus “pop” out from its complex environment (Van der Burg et al., 2008), hence the name of the effect. A common underlying circuitry for attention is the most probable cause of this effect. However, the priming of attention by means of multi-sensory information is still badly understood.

In this context of multi-sensory processing, the role of working memory cannot be discarded. Depending on the task, a simple modal representation or a more complex binding of several features at once is activated leading to a performance benefit in memorizing multi-modal (audiovisual) stimuli compared to uni-modal stimuli (Quak et al., 2015). Moreover, working memory and attention appear as two aspects of the same process (Cowan, 2001; Kiyonaga and Egner, 2013; Quak et al., 2015). Working memory could be regarded as attention directed internally, whereas selective attention to stimuli is directed externally (Cowan, 2001; Kiyonaga and Egner, 2013; Vetter et al., 2014; Talsma, 2015).

Both concepts, the priming of attention by means of multi-sensory information and working memory, are heavily mediated by individual differences. In working memory capacity the individual differences have been linked to individual differences in the susceptibility to auditory distraction in a wide variety of tasks and contexts (Sörqvist, 2010; Sörqvist and Rönnberg, 2014). However, the differences vary considerably, from slight facilitation from a noisy background to severe disruption (Ellermeier and Zimmer, 1997; Sörqvist, 2010). These findings reflect individual differences in the ability to control attention and avoid distraction (Conway et al., 2001). For instance, individuals with a high working memory capacity are less distracted by auditory distractors (Sörqvist and Rönnberg, 2014), and attention and working memory capacity can explain individual differences in the perception of audiovisual stimuli (Sun et al., 2018).

The participant's bias for either auditory or visual memory (Repp and Su, 2013), as well as their previous task experience (Iversen et al., 2015), strongly influences the expected overall benefit of auditory support in visual tasks. A follow-up study on the “pip and pop” effect indeed revealed that participants with a strong multi-sensory interaction benefit the most from the audiovisual targets, but also suffered the most from audiovisual distractors (Van der Burg et al., 2011).

Also rhythmic stimulation might enhance the “pop” effect as it enhances temporal prediction and thus opens a window for attention based on the rhythm (Jones, 1976; Large and Jones, 1999). According to the dynamical attending theory (DAT), when tone sequences are presented in a regular rhythm they entrain attentional oscillation in the brain, which facilitates the processing of information presented in phase with the rhythm (Jones, 1976; Large and Jones, 1999; Jones et al., 2002). In terms of the predictive coding theory, (auditory) regularities in the sensory input stream build up predictions that facilitate the processing of new incoming (visual) information (Rao and Ballard, 1999). The theory has also been applied to explain how rhythm is processed in the brain (Vuust and Witek, 2014). Studies have shown that auditory rhythms outperform visual ones (Repp and Su, 2013; Iversen et al., 2015), indicating that audition is generally superior to vision in terms of temporal priming (Jäncke et al., 2000; Hove et al., 2013). However, the effectiveness also depends on the modality-specific expertise of the participants. Musicians tend to be more distracted by auditory stimuli than by visual stimuli, while the opposite is true for visual experts (Hove et al., 2013).

Several hypotheses back up the idea of a dominant modality in multi-sensory processing. The modality appropriateness hypothesis describes that the more appropriate modality for the specific task dominates (Welch and Warren, 1980). For example, audition would dominate vision in a task about temporal discrimination whereas vision would dominate audition in a task about spatial orientation. The information reliability hypothesis suggests that the dominant modality is defined mainly by the reliability of the modality (Schwartz et al., 1998). The discontinuity hypothesis states that the discontinuous modality (e.g., a cello plucking video Saldaña and Rosenblum, 1993) influences the continuous modality (e.g., a bowing video Saldaña and Rosenblum, 1993) more strongly than vice versa (Shams et al., 2002). Finally, also task instructions have an effect. The directed attention hypothesis claims that the modality where attention is directed to, will be dominant (Warren, 1979).

To sum up, there is a general agreement that attention, working memory and multi-sensory processing cannot be separated from each other (Quak et al., 2015). However, their joint study seems to be very challenging, given dependencies of participant-related modality dominance, stimuli-related modality dominance and associated experiences and training. Typically there are a number of variables and confounds, as well as considerable variability in data to be dealt with, not to speak about subtle dependencies among variables. In this work, an experiment was designed that explores both the differences between people and the benefit of rhythmic sound support on performance in a task requiring visual attention. For this purpose, a study was set up involving auditory and visual stimuli and their interaction. These stimuli were used as attractors and distractors of attention and were put in sequences that require working memory in order to be memorized and recalled. The present study is based on the hypothesis that working memory and attention are closely linked processes. Therefore, our hypothesis is that sequence recall, which requires working memory, is improved by auditory support. Besides this, there are several reasons to expect that the effect will be different for non-rhythmic and rhythmic sounds. This hypothesis is supported by the dynamical attending and predictive coding theories. They state that audio cues, and certainly rhythmic audio cues, open up windows of attention that facilitate working memory and recall. This supports the assumption that a visual stimulus presented in a window of attention will get noticed as a target stimulus that enters working memory and the subsequent recall. Based on literature, it is furthermore expected that task performance is significantly affected by differences between individuals, in particular regarding audiovisual dominance and musical training. The manuscript continues with an in-depth discussion of the methodology after which results are presented and discussed in a final section.

## 2. Materials and Methods

### 2.1. Experimental Design Background

This paper's hypotheses can be tested by means of a memory task in which visual target stimuli are presented among distractor stimuli. Depending on condition, the visual targets are either non-supported, rhythmically supported without audio, non-rhythmically supported by audio or rhythmically supported by audio. Participants have to recall the sequence of targets, based on which a correctness score can be calculated as response variable. As a visual task that requires attention, a variant of the digit-span test showed to be a valuable option for the experimental design. The advantage of a digit-span test is that it cannot be affected by semantic associations (Jones and Macken, 2015). Using numbers instead of letters or words, less meaning can be associated with random sequences of symbols. Also other external factors such as appearance in daily life or complexity do not affect performance (Jones and Macken, 2015). Digit span is known to be affected by an individual's age. With increasing age, the working memory storage and processing ability decreases (Schroeder, 2014). Generally, this is attributed to a diminished ability to inhibit distractions and to focus on stimulus cues (Hills et al., 2014). The digit span task is also a common component in IQ tests such as the Wechsler Adult Intelligence Scale (Drozdick et al., 2018) and thus inherently linked to intellectual abilities. The age effect and the intelligence effect were controlled for by limiting the participants' age range to young adults under 30 and by only accepting participants if they were currently enrolled in some form of higher education or they had successfully finished it. This range also limits the variability with age in the width of the Temporal Binding Window (TBW), which reflects the audiovisual temporal integration ability of a person (Zhou et al., 2020). On average, normal healthy adults have a digit span between five and nine. This range is also known as *the magical number seven, plus or minus two* (Miller, 1956). As the aim of this study is not to measure digit span by itself, the length of the sequences is fixed at five target digits. At the lower edge of the mentioned range, a sequence of five digits should be rather easy to remember for a healthy young adult. The task was modified by including distractors in between the target stimuli, and although the goal is to remember five digits, ten digits will be presented to the participant.

Besides the length of the sequence, also the duration of the individual target and distractor digits needs to be considered. In our experiment, this is critical because a distractor will need to be inserted between the targets while the repetition rate of stimuli needs to remain compatible with common rhythm intervals. Stimulus repetition rates of the order of 50 ms may be suppressed by the “attentional blink” (Raymond et al., 1992). Recent work on identifying visually presented numbers via brain computer interfaces, showed that performance degraded once the stimulus presentation rate decreased below 100–200 ms (Lees et al., 2019). As a compromise, digits are visible on the screen for only 200 ms. A pilot study showed this to be an acceptable stimulus duration.

Target digits were presented in black, while distractor digits were presented in dark gray avoiding as many emotional meanings of color as possible (Mikellides, 2017). The exact gray color was determined on an individual basis and was set such that the difference between the black and gray digits was just noticeable. The just noticeable visual difference between targets and distractors makes the digit span task more challenging and should allow to observe the effect of added support on attention more easily.

In order to be able to perceive the rhythmic support, the stimuli have to be presented in the optimal range of human rhythm perception. Typical digit span tasks are presented at a rate of about one digit per second. Walking is known to be intrinsically rhythmic and operates around 120 steps per minute, or a step frequency of ≈ 2 Hz (MacDougall and Moore, 2005). In the large body of tapping research, a preferred tempo of ≈ 1.5–2 Hz is reported, with an optimal temporal precision within the range of 0.8–2.5 Hz (Moelants, 2002; McAuley et al., 2006; Repp and Su, 2013). Although the perceptible range of rhythms lies between 0.6 and 3.8 Hz (Woodrow, 1951; Fraisse, 1956), the major part of periodic temporal attention studies are designed with rhythms in the 1 - 2 Hz range (Zalta et al., 2020). Differences across modalities exist with the auditory periodic temporal attention operating around 1.5 Hz and visual periodic temporal attention around 0.7 Hz (Zalta et al., 2020). As a trade-off between all considerations, the interval was chosen at 1.25 s, and thus the frequency as 0.8 Hz. This time interval can accommodate the presentation of one target and one or two distractor stimuli.

Questionnaires and additional tests are included in the experiment to identify personal characteristics. General demographic information (age and gender) and information about the musical background (education, practicing musician) have been polled for. In addition, noise sensitivity and preferred/dominant modality in audiovisual perception were assessed, as these could influence the task performance and its interaction with auditory support, rhythmic or not. Electroencephalography (EEG) and magnetoencephalography (MEG) have associated noise sensitivity as a stable personal factor that is observable in everyday live with underlying neural attention and gating structures (Kliuchko et al., 2016). One possible strategy to assess noise sensitivity is by means of the Weinstein Noise Sensitivity Scale (NSS) (Weinstein, 1980), which is considered as the most well-researched and widely used measure of noise sensitivity (Benfield et al., 2014). The scale consists of items that mostly express attitude toward noise in general and emotional reactions to everyday environmental sounds. A shorter, five item questionnaire of the NSS, the Noise Sensitivity Scale Short Form (NSS-SF), was developed and proved to be reliable and representative of the original (Benfield et al., 2014). Considering the inter-individual differences in the interaction of the visual and auditory modality, previous work by our group showed a variation in audiovisual aptitude between persons that affected the interaction between appraisal of the soundscape and landscape in everyday life (Sun et al., 2018) and affected perceived noise annoyance. Their work used an audiovisual deviant detection task on everyday scenes to quantify this aptitude, but samples too many mechanisms simultaneously to be applicable here. A second possible test deals with audiovisual fission and fusion illusions (Shams et al., 2000, 2002; Andersen et al., 2004) where modality dominance is supported by different hypotheses. This test was piloted but was abandoned afterwards because it does not provide any conclusion about the auditory compared to the visual modality. The classical experiment by Giard and Peronnet (1999) was concluded to be more appropriate. Their test requires participants to correctly classify two randomly presented objects, A and B, devised according to the rules for multi-sensory integration (Stein and Meredith, 1993). Using the shortest reaction time (RT) as criteria, participants could be subdivided into two groups according to their dominant modality to perform the task: VIS-participants (shorter RTs for uni-modal visual object recognition) and AUD-participants (shorter RTs for uni-modal auditory object recognition). As it can be expected that VIS-participants experience less benefits from added audio in performing a visual task requiring attention, the test was included as part of this paper's experimental design.

### 2.2. Participants

Forty-two young adults participated in the experiment, one was excluded due to a lack of valid data. Analysis was performed with a total of 41 (*N* = 41) participants (20 females, 21 males; mean age = 23.71 ± 2.69 years). Participants were recruited through the university network and through the social and professional network of the authors. All of them declared to have normal or corrected to normal vision. Their hearing status was assessed by means of standard pure tone audiometry (PTA), using an officially calibrated *Interacoustics Clinical Computer Audiometer, model AC5* in a quiet room. All 41 participants showed normal hearing (< 25 dBHL) for the frequencies used in the experiment. In addition, participants were required to be enrolled in or finished some form of higher education. The experiment was approved by the Ethics Committee of Ghent University (Faculty of Arts and Philosophy) and informed written consent was obtained from each participant. After completing the experiment, all participants received a €20 gift voucher as compensation for their participation.

### 2.3. Questionnaires

After being acquainted with the experiment and signing the informed consent, participants were presented with a series of questions. Those polled for general demographic information, as well as background information on education, (past) musical training and being an active practicing musician. The emotional state of the participants was surveyed by asking the participants to indicate their current mood with a cross on a blank valence-arousal chart (Russell, 1980). The position of that cross with respect to the axes of the chart is reflected in a value for valence and for arousal. Additional questions served to confirm participation requirements, checked for possible underlying influences on attention (learning difficulties, attention disorders, drug usage, caffeine consumption), polled for their weekly physical activity, asked how tired participants are and how good they are in remembering digit sequences like telephone numbers or pin. The questionnaire also included the NSS-SF (Weinstein, 1980; Benfield et al., 2014), but with a zero (totally disagree) to 10 (totally agree) response scale (Aletta et al., 2018). After completion of the experimental tasks, three additional short questions about the participant's perception of the main experiment were asked: whether they perceived the sound/rhythm as distracting or as supporting and how difficult they found the task.

### 2.4. Experimental Setup

For the duration of the experimental procedure, participants were seated in a comfortable chair right in front of a small display (Nikkei NLD20MBK, 20 inch (51 cm), 50 Hz) which was positioned at eye-height and at a viewing distance of 1.5 m. The refresh rate of this screen was fast enough to accommodate the 200 ms stimuli and assure synchronization between auditory and visual stimuli. Sounds were delivered from the computer to a soundcard (RME Fireface UCX). One audio channel contained the sound stimulus, while the other audio channel contained a trigger signal for synchronization purposes. The stimulus channel was duplicated so that the same stimulus could be presented at each ear. All sounds were played at a calibrated level of 70 dBSPL and delivered through ER-2 insert earphones (Etymotic Research). The experiment was conducted in a soundproof and electromagnetically shielded booth. Soft spots with a warm color provided enough light to perform the task in enjoyable lighting conditions.

### 2.5. Audiovisual Dominance Pre-test

**Figure 1:**
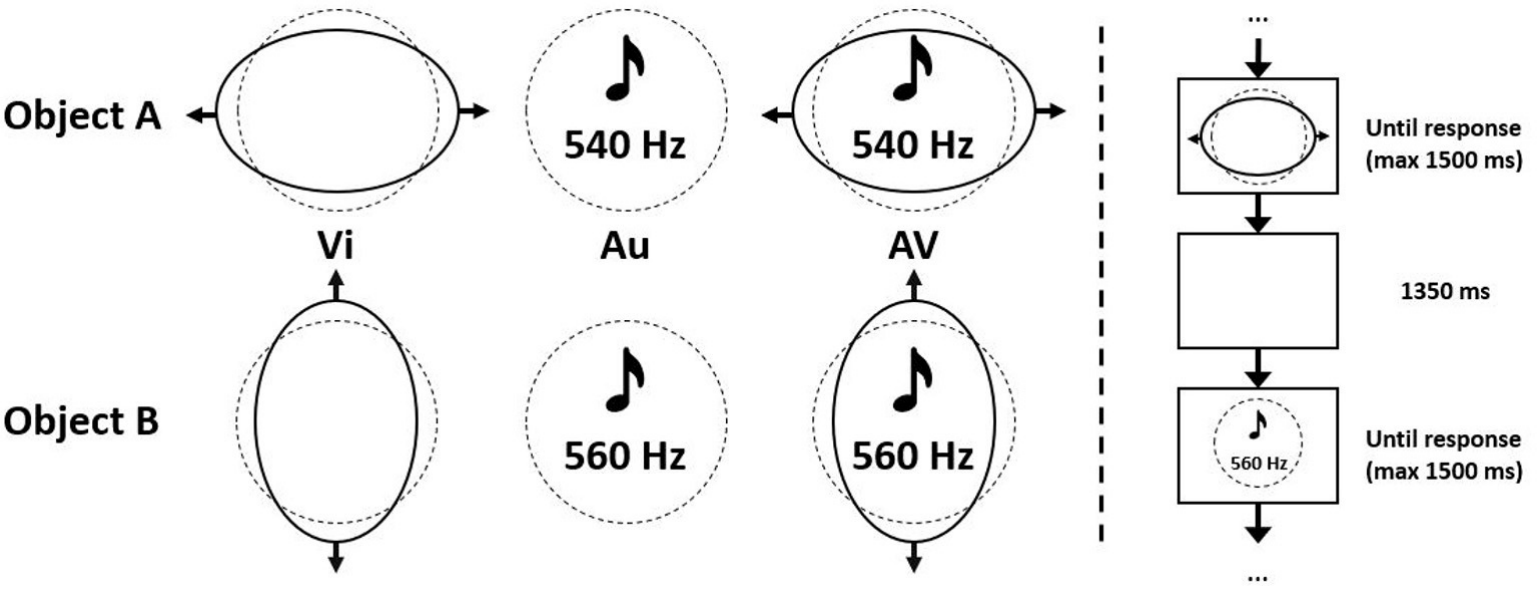
Stimulus details of the audiovisual dominance task, including characteristics of objects A and B. Created based on Giard and Peronnet (1999).

The audiovisual dominance test, as mentioned earlier, is based on an object recognition task by Giard and Peronnet (1999). Participants were randomly presented with two objects, A and B, and were asked to correctly classify these objects as fast as possible by pressing the left or down arrow key, corresponding to object A and B, respectively. Objects were defined by visual (Vi) features alone, auditory (Au) features alone or the combination of visual and auditory features (AV). The visual part of the object consisted of a circle deforming into an ellipse, either horizontally (object A) or vertically (object B). The auditory part consisted of a tone of 540 Hz (object A) or 560 Hz (object B) (Figure 1). This combines in six different stimuli to be shown. All trials were presented using the PsychoPy presentation software (Peirce et al., 2019) in such a way that at most, but usually less than, four identical stimuli could succeed each other. In total 108 trials were presented, from which the first 12 (two of each stimulus) were considered as practice trials and not concluded in further analysis. After onset, stimuli were visible until a response was received, unless this took longer than 1.500 ms. Stimulus offset and the next stimulus onset are separated by a fixed interval of 1.350 ms (Figure 1). For every trial, reaction time (RT) and response correctness were recorded. All trials with a wrong, or slower than 1.500 ms, response were removed from analysis. A dataset was constructed, including per participant the mean reaction time in the auditory, the visual and the audiovisual condition. Consequently a k-means (k = 2) cluster analysis was performed on this dataset. After assigning a meaning to both clusters, 21 participants could be considered as auditory dominant and 20 participants as visually dominant. This newly created variable, *Dominance*, is used as an input variable in the analysis of the main experiment.

### 2.6. Main Experiment

**Figure 2:**
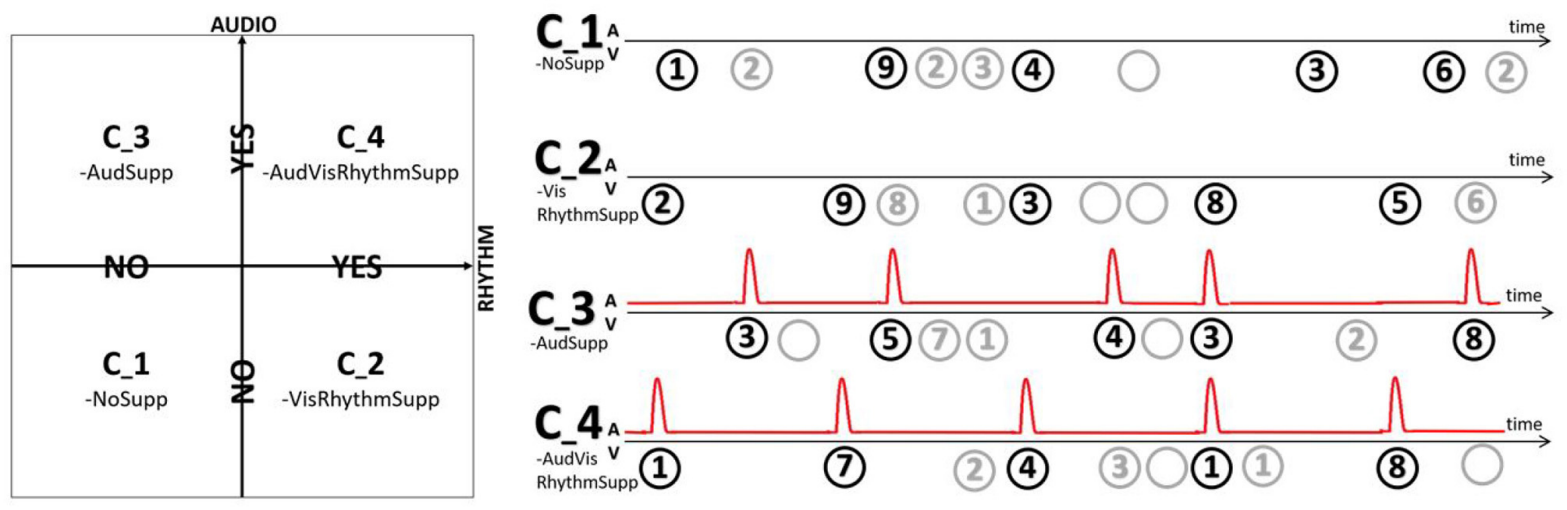
Depending on the condition either no support (C_1-NoSupp), visual rhythmic support (C_2-VisRhythmSupp), auditory non-rhythmic support (C_3-AudSupp) or both auditory and visual rhythmic support (C_4-AVRhythmSupp) was provided.

The main part of the experiment consisted of a modified digit span task. A target digit was presented, followed by either no, one or two distractor digits, another target, no, one or two distractor digits, etc. (Figure 2). Targets and distractors were presented such that every sequence always consisted of five target and five distractor digits. At the start and end of every sequence a fixation cross was included. Both target and distractor digits were presented as an encircled number in the range one to nine, all with equal probability. To further reduce the predictability and increase the distractive nature, also blanks were included as a possible distractor digit. Encircling them, and thus presenting them as empty dark gray circles, assured they are distinguishable from showing nothing. Across the experiment, four conditions were compared:

- C_1-NoSupp: visual targets presented non-rhythmically.
- C_2-VisRhythmSupp: visual targets presented rhythmically.
- C_3-AudSupp: visual targets presented non-rhythmically, synchronized non-rhythmic audio support.
- C_4-AVRhythmSupp: visual targets presented rhythmically, synchronized rhythmic audio support.

Thirty different sequences were created, ensuring that succeeding target digits were always different (e.g., 12234 is not possible, but 12324 is). Recognizable target patterns were manually identified and excluded (e.g., 12345, 12121, 12468, etc.). The value of the distractors in between was completely random. These 30 sequences were shown in four different conditions, leading to a total of 120 sequences. Every participant was shown the same sequences, but in a different order. The order of the presentation conditions and sequences was pseudo-randomized in such a way that the current condition was different than the previous one and such that at least two other sequences were presented before a previous one was repeated. Targets were depicted in black, *rgb*(0, 0, 0). Distractors were visualized in dark-gray, *rgb*(*x, x, x*), *x* ∈ (50, 75), with the exact value of *x* assessed individually (see Section 2.1). After every 15 sequences, there was by default a small 10 s break to relax. Upon request, breaks could be elongated. Most participants relished one or two longer breaks.

In conditions C_3-AudSupp and C_4-AVRhythmSupp, the targets were presented in synchrony (same onset) with an auditory support. This was a 500 Hz tone burst, 50 ms long with 5 ms fade in and 5 ms fade out. In conditions C_2-VisRhythmSupp and C_4-AVRhythmSupp, targets were presented in synchrony with an underlying beat, meaning they had a inter-stimulus (target onset to next target onset) interval of exactly 1.25 s (0.8 Hz). In these rhythmic conditions, the digit sequence was preceded by five induction stimuli with the same underlying beat to induce the participant with the rhythm. For C_2-VisRhythmSupp this was done solely visual, by means of empty black circles. Induction in C_4-AVRhythmSupp was performed only auditory, by means of synchronized tone bursts. In the non-rhythmic conditions (C_1-NoSupp and C_3-AudSupp), targets were presented at a variable rate, with at most one target digit in every 1.25 s time window and taking into account the amount of distractors for that target. The distractors were always played back at a random moment, within their allowed time interval.

Although there are only five targets, participants were instructed to “remember the five to seven black target digits.” This ensured participants could not predict the end of the sequence and kept focusing until the end. They were made aware that the first digit always was a target digit shown in black. No special mention was made about the audio or rhythm, in order not to draw attention to it. After the sequence, the fixation cross disappeared for 7 s, during which the participants orally recalled the remembered sequence. This response was recorded and afterwards manually transcribed by the experimenter.

### 2.7. Outcome Scores

The digit recall accuracy is assessed from different angles. An error can occur due to lack of focused attention to the environment triggered by the multi-sensory stimuli, but it can also occur during remembering and recall. Hence, several error scores are evaluated, each of them being more sensitive to specific sources of error.

- The PerDigit score measures the correctness of the answer by individually comparing every digit with the corresponding digit in the correct answer. In case the answer and target sequence are different in length, dummy digits are inserted before comparison. For instance, the target sequence from Figure 2, C_1-NoSupp is “19436.” By extending a given answer “1946” to “194*6,” there are four out of five digits in the correct position. Re-scaling this to the interval [0, 1] results in a PerDigit score of 0.8. The other way around, if the answer is “129436”, the correct answer sequence can be extended to “1*9436” to achieve a score of five out of six, resulting in the PerDigit score 0.83.
- The Levenshtein score is the distance between two sequences, which corresponds to the minimal number of operations needed to go from one sequence to another (Levenshtein, 1966). Allowed operations are the insertion or deletion of one single digit, or the substitution of one digit with another one.
- The Distraction score was designed to reflect to what extent participants are distracted by the presence of the distractor digits and remember them, rather than remembering the targets. This score more directly assesses the influence of the effect of auditory support (rhythmic or not) on attention focusing and is expected to be less influenced by working memory than the previous scores. Groups of three digits in the answer sequence, with special cases for the start and the end, are compared to the target sequence and to the combined target and distractor sequence. The number of groups corresponding with the target is than subtracted from the number of groups corresponding with the combined target and distractor sequence. Retake our example from Figure 2, C_1-NoSupp (1_2_9_23_436_2_, the blank distractor digit already removed) and assume the answer given is “19436”. The first group of three digits of the answer sequence (“194”) is found in the target sequence, but not in the target and distractor sequence. The same holds for the second three digit group (“943”). The third group (“436”) is found in both. As special cases also the start (“19”) and the end (“36”) of the sequence are checked. Subtracting the number of groups corresponding with the target (= 5) from the number of groups corresponding with the target and distractor sequence (= 1) and rescaling this result (= –4) based on the length of the answer (= 5) gives the final Distraction score (= –0.8). This means that the participant was less distracted by the distractors than attracted by the targets, thus the negative score. As an opposite example, consider the sequence 29_81_385_6_, again with the blank distractors already removed, and assume “29856” as the answer. Following the same procedure a Distraction score of (4 - 1)/5 = 0.6 is achieved. Because the maximal number of three digit groups plus the two edge cases is always equal to the length of the answer, all scores fall in the range [–1, 1], which corresponds to [not distracted at all, very distracted].

### 2.8. Statistics

Statistic testing was performed by means of the Kruskal-Wallis test because it is considered to be the non-parametric alternative to the one-way ANOVA. While the latter assumes normality, Kruskal-Wallis does not require it and is therefore preferred for this research's data. In line with the choice for the non-parametric Kruskal-Wallis test, pairwise *post-hoc* testing was done by means of the non-parametric Wilcoxon signed rank test because in every condition the same sequences are measured and compared and are thus considered paired samples.

This work's hypotheses are validated by constructing statistical models to predict each of the three outcome scores based on a number of independent variables and random variables. The variables used in the models are either experiment variables that define the course of the experiment or variables related to the personal characteristics of the participant. Personal variables are linked to the responses given in the questionnaires.

- “Participant” represents the participant. It is the only variable modeled as a random effect because there are way more possible participants than those that participated in the experiment.
- “Condition” points to the four presentation conditions and is denoted as “C_X” or “C_*X-example*.”
- “Order” reflects at what moment in time the sequence was presented in the experiment (a rank between 1 and 120).
- “Sequence” is a number between 1 and 30 to identify every sequence of digits.
- “MusActive” and “MusEducation” indicates whether or not the participant had some musical training, based on the questionnaire response. It reflects regular music practice and music education of some sort, respectively.
- “Dominance” is the result of the audiovisual dominance pre-test described earlier.
- “Sex,” “Age,” and “Handedness” are self-explanatory.
- “M-Valence” and “M-Arousal” represents the participants mood on both axes of the valence-arousal chart, as explained previously.
- “Activity” represents physical activity per week, assessed on a five-point scale (less than once, 1 to 2, 3 to 4, and 5 to 6, more than 6 times per week).
- “Caffeine” indicates whether or not the participant consumed any form of caffeine prior to the experiment.
- “NoiseSensitivity” is the result of a 2 cluster analysis on the NSS-SF, resulting in either tolerant or sensitive to noise.
- “PercTiredness,” “PercMemoryDifficulty,” and “PercTaskDifficulty” are variables assessed on a six-point scale. They reflect how tired one feels prior to the experiment, how difficult they find it to remember sequences of numbers (e.g., telephone numbers or pin codes) and how difficult they perceived the main task.
- “PercRhythmSupport” and “PercSoundSupport” express to what level the rhythm and sounds were perceived as disturbing (lower values) or supporting (higher values).

Cumulative link (mixed) models (Christensen, 2019) are used for the prediction modeling, so that data can be predicted by means of the parameters in a model. These are also known as ordered regression models, proportional odds models and ordered logit/probit models. Separate models were built for the PerDigit score (PD), the Levenshtein score (L) and the Distraction score (D). The scores were all modeled as ordinal response variables, because they clearly contain a natural ranking, but equal increments in the scores do not necessarily represent equal increments of the underlying attribute. The analysis was performed based on the multilevel approach. The first level contains all variables that change over the course of the experiment, alias the experimental variables. The second level includes all other variables ($\underset{k}{\sum }Z_{jk}$), which don't change during the experiment and are all related to the personal level. The parameter *u*_*ij*_ reflects the random effect, in this case the variable Participant. Equations (1 to 5) show the full theoretical multilevel model. No random effects were added in Equations 3 and 4 because no interaction is expected between Participant and Condition or between Participant and Sequence. This assumption was later confirmed in model testing. An iterative approach was taken to simplify the models variable by variable, as described in Section 3.3. Model comparison and selection was performed mainly based on the Akaike Information Criterion (AIC) (Akaike, 1974). When comparing the AIC of two models, the one with the lower AIC was considered as the better one if it was significantly different based on an analysis of variance (ANOVA) likelihood-ratio test (Chambers and Hastie, 1992). Reported *p*-values are Chi-Squared values. If models were not significantly different, the decision was made based on the significance of the individual variables in the model and on the number of variables in the model.


(1) $$\begin{array}{l} \text{Level }1\;(\text{Time}):\\ \;Y_{ij}=\beta_{0j}+\beta_{1j}\;Condition_{ij}+\beta_{2j}\;Sequence_{ij}\\ \;+\beta_{3j}\;Order_{ij}+r_{ij} \end{array}$$

(2) $$\begin{array}{l} \text{Level }2\;(\text{Person}):\\ \;\beta_{0j}=\gamma_{00}+\sum_{k}\gamma_{0k}\;Z_{jk}+u_{0j} \end{array}$$

(3) $$\begin{array}{l} \beta_{1j}=\gamma_{10}+\sum_{k}\gamma_{1k}\;Z_{jk} \end{array}$$

(4) $$\begin{array}{l} \beta_{2j}=\gamma_{20}+\sum_{k}\gamma_{2k}\;Z_{jk} \end{array}$$

(5) $$\begin{array}{l} \beta_{3j}=\gamma_{30}+\sum_{k}\gamma_{3k}\;Z_{jk}+u_{3j} \end{array}$$

## 3. Results

### 3.1. Distributions of the Outcome Scores

**Figure 3:**
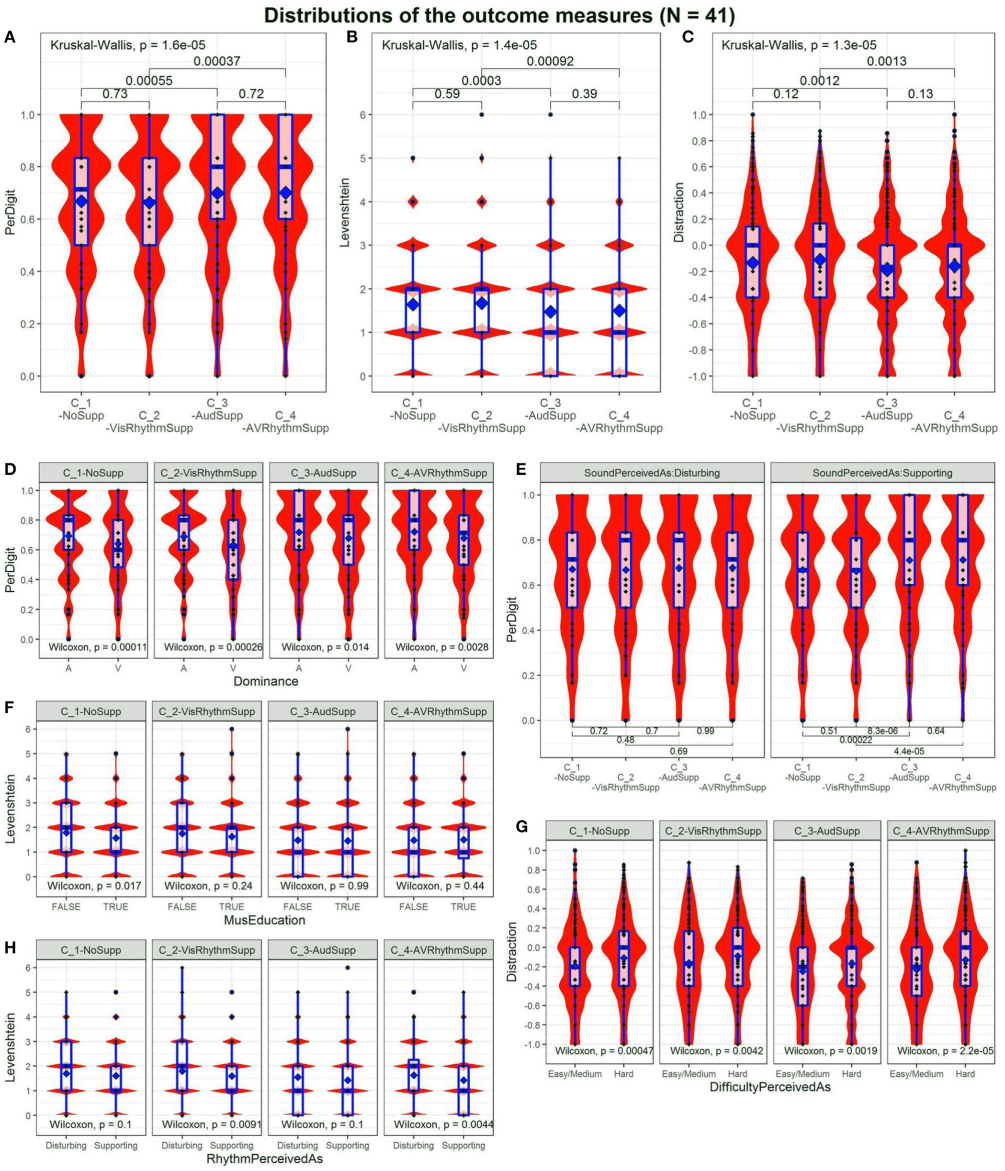
Distributions for the different outcome scores across conditions. Significance *p*-values between conditions are indicated. **(A)** “PerDigit” score. **(B)** “Levenshtein” score. **(C)** Distraction score. The bump at –0.4 is due to the way scores are calculated. **(D)** Influence of audiovisual Dominance on task performance across conditions. **(E)** Influence of SoundSupport (Disturbing vs. Supporting) on task performance. **(F)** Influence of MusEducation on task performance across conditions. **(G)** Influence of TaskDifficulty (Easy/Medium vs. Hard) on task performance across conditions. **(H)** Influence of RhythmSupport (Disturbing vs. Supporting) on task performance across conditions.

Figures 3A–C show the distributions of the outcome scores. Figure 3A shows the PerDigit score distribution. Because most responses consist of five digits, scores are concentrated around ^0^/_5_, ^1^/_5_, ^2^/_5_, ^3^/_5_, ^4^/_5_ and ^5^/_5_. Other data points correspond with responses that were longer or shorter. Overall, there is a significant difference across conditions (Kruskal-Wallis, *p* = 1.6e-05). *Post-hoc* testing shows that C_3-AudSupp is significantly better than C_1-NoSupp (Wilcoxon, *p* = 5.5e-04) and C_4-AVRhythmSupp is significantly better compared to C_2-VisRhythmSupp (Wilcoxon, *p* = 3.7e-04) while C_1-NoSupp is not significantly different from C_2-VisRhythmSupp (Wilcoxon, *p* = 0.73) and C_3-AudSupp is not significantly different from C_4-AVRhythmSupp (Wilcoxon, *p* = 0.72).

Figure 3B displays the Levenshtein score distribution. It peaks at integer values as these are the only possible values this score can take. There is an overall significance across conditions (Kruskal-Wallis, *p* = 1.4e-05). *Post-hoc* testing shows that C_1-NoSupp and C_2-VisRhythmSupp are not significantly different (Wilcoxon, *p* = 0.59), just as C_3-AudSupp and C_4-AVRhythmSupp are not (Wilcoxon, *p* = 0.39). Following the same trend as the PerDigit score, C_1-NoSupp and C_3-AudSupp are significantly different (Wilcoxon, *p* = 3e-04) and so are C_2-VisRhythmSupp and C_4-AVRhythmSupp (Wilcoxon, *p* = 9.2e-04).

Figure 3C shows the Distraction score distribution, where again the same conclusion can be drawn. There is an overall effect of conditions (Kruskal-Wallis, *p* = 1.3e-05). Conditions with audio have a significantly lower value compared with those without audio: C_3-AudSup*p* < C_1-NoSupp (Wilcoxon, *p* = 1.2e-03) and C_4-AVRhythmSup*p* < C_2-VisRhythmSupp (Wilcoxon, *p* = 1.4e-03). Conditions with rhythm are not significantly different from their non-rhythmic counterparts: C_1-NoSupp vs. C_2-VisRhythmSupp (Wilcoxon, *p* = 0.12) and C_3-AudSupp vs C_4-AVRhythmSupp (Wilcoxon, *p* = 0.13). It might seem remarkable that the distributions for the Distraction score show two maxima, one around 0.0 and one around –0.4 but calculating the score on prototype answers shows this is due to the way the Distraction score is calculated.

Overall, the analysis of distributions of the outcome scores reveals a significant effect of sound on all of the scores while there is no significant effect of rhythm.

### 3.2. Conditional Dependencies

Figures 3D–H reveal dependencies on some individual differences of interest. The panels are representative for all three outcome scores. In the post-test questionnaire, participants who reported that they perceived the sound as slightly to very much disturbing show no significant performance difference between conditions, whereas those that experienced the sound as slightly to very much supporting show an increased performance in conditions C_3-AudSupp and C_4-AVRhythmSupp on all three performance scores (Wilcoxon; C_1-NoSupp vs. C_3-AudSupp, PD: *p* = 2.2e-04, L: *p* = 8.1e-05, D: *p* = 4.9e-04; C_2-VisRhythmSupp vs. C_3-AudSupp, PD: *p* = 8.3e-06, L: *p* = 2.8e-06, D: *p* = 1.1e-06; C_2-VisRhythmSupp vs. C_4-AVRhythmSupp, PD: *p* = 4.4e-05, L: *p* = 1.7e-04, D: *p* = 8.9e-04). In case of the PerDigit score, this is presented in Figure 3E. These results indicate that participants are fully aware of the fact that they were helped by the sound.

Participants that stated they perceived the rhythm as disturbing as opposed to supporting, only show significantly worse performance in C_2-VisRhythmSupp (Wilcoxon, PD: *p* = 0.011, L: *p* = 9.1e-03, D: *p* = 9.8e-03) and C_4-AVRhythmSupp (Wilcoxon, PD: *p* = 6.6e-03, L: *p* = 4.4e-03, D: *p* = 1.9e-03). This result is quite logical because conditions C_2-VisRhythmSupp and C_4-AVRhythmSupp involve rhythm. Those who find rhythms supporting thus have a better result on the Levenshtein score. Remarkably, persons stating that the rhythm was supportive show a considerable (*p* < 0.11) better performance across conditions for the PerDigit (better = higher) and Levenshtein (better = lower) scores. The Levenshtein score conditioned by how rhythm is perceived, is shown in Figure 3H.

Checking whether or not a music education changes the performance in specific conditions, a significant difference is only obtained for C_1-NoSupp in case of the Levenshtein score (Wilcoxon, L: *p* = 0.017). In case of the PerDigit score, C_1-NoSupp shows a very weak significant difference (Wilcoxon, PD: *p* = 0.054). The Distraction score showed no considerable differences at all. Considering all outcome scores, a weak trend can be observed: participants with a music education perform (slightly) better in C_1-NoSupp, while in C_4-AVRhythmSupp the better performance seems to be for those without music education. Figure 3F gives an impression of the Levenshtein score conditioned by music education.

The audiovisual dominance, as established in the audiovisual dominance pre-test, divides the participant pool into auditory and visually dominant participants. The latter group performs significantly worse in all four conditions (Wilcoxon; C_1-NoSupp, PD: *p* = 1.1e-04, L: *p* = 6.2e-06, D: *p* = 2.7e-04; C_2-VisRhythmSupp, PD: *p* = 2.6e-04, L: *p* = 9.8e-05, D: *p* = 1.2e-03; C_3-AudSupp, PD: *p* = 1.4e-02, L: *p* = 5.0e-03, D: *p* = 2.2e-02; C_4-AVRhythmSupp, PD: *p* = 2.8e-03, L: *p* = 1.2e-03, D: *p* = 7.7e-03). Figure 3D shows the difference in behavior between auditory and visual dominance.

Finally, across all conditions scores were significantly worse for those that rated the task as rather difficult (Wilcoxon; C_1-NoSupp, PD: *p* = 1.9e-08, L: *p* = 6.3e-08, D: *p* = 4.8e-04; C_2-VisRhythmSupp, PD: *p* = 1.2e-06, L: *p* = 4.7e-06, D: *p* = 4.3e-03; C_3-AudSupp, PD: *p* = 1.2e-05, L: *p* = 1.2e-04, D: *p* = 1.9e-03; C_4-AVRhythmSupp, PD: *p* = 1.7e-04, L: *p* = 5.6e-04, D: *p* = 2.3e-05). An example of this behavior in case of the Distraction score is displayed in Figure 3G.

To sum up, the distributions of the outcome scores reveal interesting differences in condition. These results are furthermore conditioned by factors pointing to between-subject differences.

### 3.3. Predictive Modeling

Predictive models are used to understand how predictor variables contribute to the outcome scores. An important aspect of the modeling is that time, by means of the variable Order, is included in the analysis. This way a possible learning effect due to the repeated measures is accounted for. A separate model was constructed for each outcome score. Given the large number of predictor variables, an iterative strategy to develop the models was adopted (Table 1). Starting from a basic model, level one and subsequently level two variables were added. To become the basic model X0, the parameters γ_00_, *u*_0*j*_ and γ_30_ from Equations 2 and 5 were substituted in the level one equation (1). This way only the random variable Participant and the time variable Order are included. This basic model has the highest AIC value. Adding the parameters γ_10_ and γ_20_, i.e., the intercepts, to model X0 led to X1. By also substituting the γ_0*k*_(*Z*_*jk*_) parameters into the equation and removing those that made no significant improvement, model X2 was achieved. In order to achieve model X3, the parameter β_1*j*_ was substituted as a whole, and thus including the slope, after which non significant variables were removed. The same procedure was followed with β_2*j*_. For the PerDigit and Levenshtein models none of the *Z*_*jk*_ improved the model, but in case of the Distraction model the variable M-Valence made a significant improvement to the model. In the same way, the remaining parameters of β_3*j*_ were included to end up with model X4. For completeness, two similar equations were substituted containing Order^2^ in one, and the interaction term Order:Condition in the other. The quadratic term could possibly reflect a change in the rate of learning, and would account for the counteracting effects of task familiarity and fatigue while the interaction term would reflect a different rate of learning depending on condition. However, even with only the intercept included, both did not improve the model and were therefore omitted. To end up with the final X5 models, the X4 models were simplified by checking what variables could be removed such that their AIC was not significantly worse.

**Table 1:** Step by step multilevel model analysis.

**Name**	**Model (PD: PerDigitBin; L: Levenshtein; D: DistractionBin)**	***AIC*_*PD*_**	***AIC*_*L*_**	***AIC*_*D*_**
PD0/ L0/ D0	~ 1 + (1∣Participant) + Order	14891.42	14424.74	19487.35
PD1/ L1/ D1	~ 1 + (1∣Participant) + Order + Sequence + Condition	14718.83	14234.54	19254.18
PD2	~ 1 + (1∣Participant) + Dominance + PercTaskDifficulty	14712.11		
	+ PercRhythmSupport + M-Valence + Order + Sequence + Condition			
L2	~ 1 + (1∣Participant) + Sex + Dominance + PercTaskDifficulty		14225.09	
	+ PercRhythmSupport + M-Valence + Order + Sequence + Condition			
D2	~ 1 + (1∣Participant) + Sex + M-Valence + Order + Sequence + Condition			19247.76
PD3	~ 1 + (1∣Participant) + Dominance + PercTaskDifficulty	14706.15		
	+ PercRhythmSupport + M-Valence + Order + Sequence			
	+ Condition : PercSoundSupport			
L3	~ 1 + (1∣Participant) + Sex + Dominance + PercTaskDifficulty		14215.39	
	+ PercRhythmSupport + M-Valence + Order + Sequence			
	+ Condition : [PercSoundSupport + MusEducation]			
D3	~ 1 + (1∣Participant) + Sex + M-Valence + Order + Sequence			19239.29
	+ Condition : TaskDifficulty			
PD4	~ 1 + (1 + Order∣Participant) + Dominance + PercTaskDifficulty	14669.54		
	+ PercRhythmSupport + Order : [M-Valence + PercTiredness]			
	+ Sequence + Condition : PercSoundSupport			
L4	~ 1 + (1 + Order∣Participant) + Sex + Dominance + PercTaskDifficulty		14146.48	
	+ PercRhythmSupport + Order : [M-Valence + PercTiredness]			
	+ Sequence + Condition : [PercSoundSupport + MusEducation]			
D4	~ 1 + (1 + Order∣Participant) + Sex + Order			19152.14
	+ Sequence : M-Valence + Condition : PercTaskDifficulty			
**PD5**	~ 1 + (1 + Order∣Participant) + PercTaskDifficulty + PercRhythmSupport	**14668.42**		
	+ Order : PercTiredness + Sequence + Condition : PercSoundSupport			
**L5**	~ 1 + (1 + Order∣Participant) + Sex + Dominance + PercTaskDifficulty		**14143.81**	
	+ PercRhythmSupport + M-Valence + PercTiredness + Order			
	+ Sequence + Condition : [PercSoundSupport + MusEducation]			
**D5**	~ 1 + (1 + Order∣Participant) + Sex + Order + Sequence			**19147.39**
	+ Condition : PercTaskDifficulty			

*The resulting and best models are marked in gray*.

All iterations between the X0 and the X4 models showed significant improvements for all three outcome scores (PD: PD0 − (*p* < 2.2e-16) − PD1 − (*p* = 5.334e-03) − PD2 − (*p* = 7.423e-03) − PD3 − (*p* = 6.823e-09) − PD4, L: L0 − (*p* < 2.2e-16) − L1 − (*p* = 1.584e-03) − L2 − (*p* = 1.179e-03) − L3 − (*p* = 1.422e-15) − L4, D: D0 − (*p* < 2.2e-16) − D1 − (*p* = 5.468e-03) − D2 − (*p* = 2.448e-03) − D3 − (*p* < 2.2e-16) − D4). Finally, model D4 is significantly improved to achieve model D5 (*p* = 3.333e-03). Model PD4 is also simplified to end up with model PD5, which is although the lower AIC not significantly better (*p* = 0.1811) but has three degrees of freedom less. Similarly, model L4 is simplified to L5, which is also not significantly better (*p* = 0.5154), but has two degrees of freedom less. The models for each outcome score improve most in terms of AIC when the experimental variables Condition and Sequence are added.

**Figure 4:**
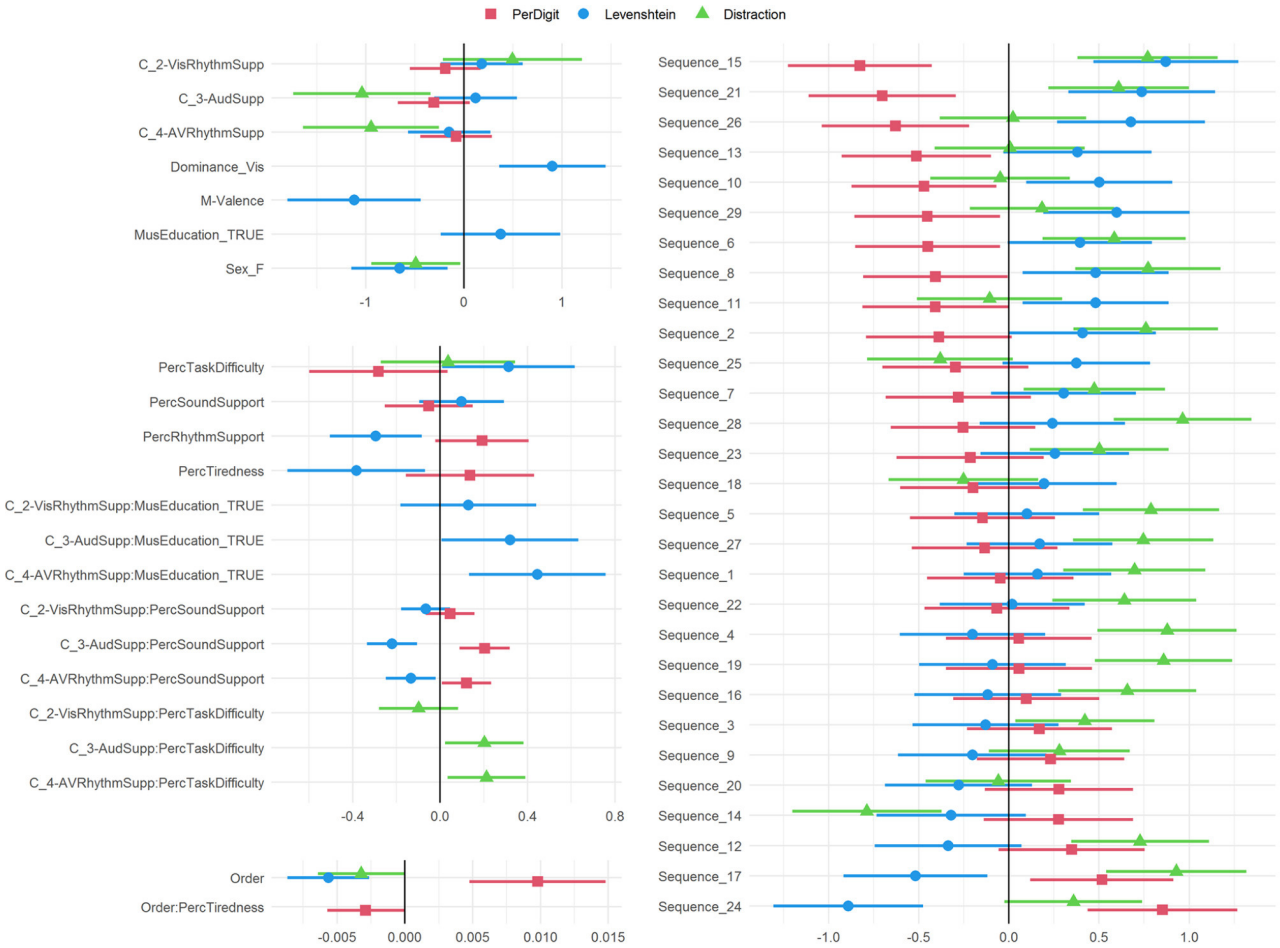
Summary of all model variables with estimates and 95% confidence intervals. If the intervals do not overlap with the zero, the corresponding variable is significantly different from the not-listed reference value. In case of continuous variables this means that a one unit change is significant.

The final models PD5, L5, and D5 are further analyzed in Figure 4, showing the coefficients in the logistic regression. Interactions between multiple variables need to be interpreted carefully and their odds ratios (OR) can be calculated for every situation based on Equations 6 and 7. The *OR*() values can be fetched from Table 2. The random effects can be summarized based on the standard deviations of the odds ratios, for both the Participant intercept and the Participant:Order interaction. Intercept: 0.70729; Order: 0.00661 for the PerDigit model; Intercept: 0.68048; Order: 0.00852 for the Levenshtein model and Intercept: 0.70022; Order: 0.00928 for the Distraction model. For our main experimental variable Condition, the *p*-value for the comparison of the four levels in this categorical variable are shown in Table 3.


(6) $$\begin{array}{l} logit\left(Y\right)=C+\alpha \;Var1+\beta \;Var2+\gamma \;Var1\;Var2 \end{array}$$

(7) $$\begin{array}{l} OR\left(Y\right)=Exp\left(logit\left(Y\right)\right)=C^{\prime }\;\left[OR{\left(\alpha \right)}^{Var1}\right]\;\left[OR{\left(\beta \right)}^{Var2}\right]\\ \;[OR{\left(\gamma \right)}^{Var1\;Var2}] \end{array}$$

For the Distractor (D) outcome score, which is expected to be most closely related to focusing attention on the target vs. the distractor, a significant reduction in the odds ratio for conditions including sound (C_3-AudSupp: *p* = 3.506e-03 and C_4-AVRhythmSupp: *p* = 7.331e-03) is observed. In addition a significant difference between these two groups of conditions with perceived task difficulty is observed: those that perceive the task as difficult have no benefit of the added sound, or stated differently, those that do not benefit from the auditory support state that the task is difficult more than others (C_3-AudSupp:PercTaskDifficulty: *p* = 2.688e-02, C_4-AVRhythmSupp:PercTaskDifficulty: *p* = 1.912e-02). Sex (female scores higher) and Order of the sequence are also included in the model. For the latter it should be noted that there is also an interaction of Order with Participant and hence the amount of improvement in task performance with time also depends on the participant.

**Table 2:** Odds ratios (OR) of all terms in the final model for all three outcome scores; OR are given per unit increase of the independent variable.

**Term**	***OR*_*PD*_**	***OR*_*L*_**	***OR*_*D*_**
C_1-NoSupp	Reference level		
C_2-VisRhythmSupp	0.82611	1.19915	1.63746
C_3-AudSupp	0.73491	1.12369	0.35242
C_4-AVRhythmSupp	0.92254	0.85933	0.38686
Dominance_Aud (21p)	Reference level		
Dominance_Vis (20p)	/	2.46331	/
M-Valence	/	0.32583	/
MusEducation_FALSE (13p)	Reference level		
MusEducation_TRUE (28p)	/	1.45056	/
Sex_M (21p)	Reference level		
Sex_F (20p)	/	0.51774	0.61138
PercTaskDifficulty	0.75428	1.36736	1.03643
PercSoundSupport	0.94932	1.10271	/
PercRhythmSupport	1.21133	0.74481	/
PercTiredness	1.14636	0.68176	/
C_2-VisRhythmSupp:	/	1.13746	/
MusEducation_TRUE			
C_3-AudSupp:	/	1.37629	/
MusEducation_TRUE			
C_4-AVRhythmSupp:	/	1.56096	/
MusEducation_TRUE			
C_2-VisRhythmSupp:	1.04749	0.93635	/
PercSoundSupport			
C_3-AudSupp:	1.22547	0.80244	/
PercSoundSupport			
C_4-AVRhythmSupp:	1.12849	0.87430	/
PercSoundSupport			
C_2-VisRhythmSupp:	/	/	0.90661
PercTaskDifficulty			
C_3-AudSupp:	/	/	1.22409
PercTaskDifficulty			
C_4-AVRhythmSupp:	/	/	1.23633
PercTaskDifficulty			
Order	1.00984	0.99438	0.99677
Order:PercTiredness	0.99711	/	/
Sequence_0	Reference level		
Sequence_15	0.43750	2.38845	2.15876
Sequence_21	0.49550	2.08907	1.83784
Sequence_26	0.53300	1.96869	1.02227
Sequence_13	0.59852	1.46259	1.00531
Sequence_10	0.62438	1.65072	0.95195
Sequence_29	0.63571	1.81781	1.20161
Sequence_6	0.63752	1.48218	1.79357
Sequence_8	0.66542	1.61676	2.16284
Sequence_11	0.66488	1.61809	0.89828
Sequence_2	0.67853	1.50574	2.13465
Sequence_25	0.74341	1.45445	0.68250
Sequence_7	0.75560	1.35409	1.60587
Sequence_28	0.77543	1.27272	2.62071
Sequence_23	0.80720	1.29067	1.65073
Sequence_18	0.81985	1.21430	0.77709
Sequence_5	0.86407	1.10491	2.19851
Sequence_27	0.87426	1.18607	2.10531
Sequence_1	0.95346	1.17271	2.00527
Sequence_22	0.93580	1.01845	1.89720
Sequence_4	1.05639	0.81763	2.40416
Sequence_19	1.05732	0.91345	2.35618
Sequence_16	1.10082	0.88994	1.92900
Sequence_3	1.18426	0.87888	1.52351
Sequence_9	1.26061	0.81679	1.32288
Sequence_20	1.32003	0.75690	0.94353
Sequence_14	1.31669	0.72624	0.45503
Sequence_12	1.41651	0.71456	2.06999
Sequence_17	1.67533	0.59547	2.53204
Sequence_24	2.34403	0.41037	1.43004

**Table 3:**
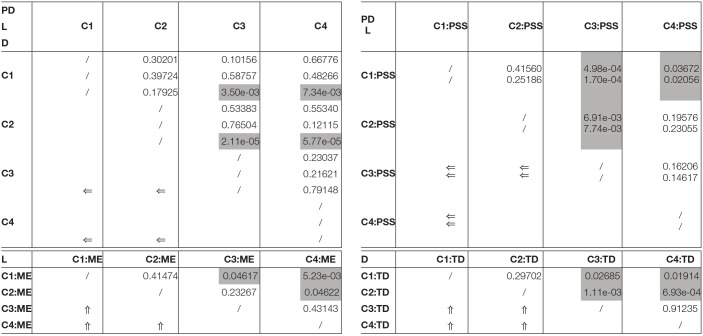
Resulting *p*-values when comparing the levels of multilevel categorical variables to each other.

*Significant p-values (p < 0.05) are marked in gray. If significant, the arrow indicates the better condition*.

The PerDigit (PD) outcome score assesses in a more holistic way the overall result of the experiment. Condition is included in the final model but significance is only obtained in combination with the perception of sound support by the participant. All support conditions (C_2-VisRhythmSupp, C_3-AudSupp and C_4-AVRhythmSupp) tend to reduce the accuracy on a per digit basis, but for people reporting perceived support by sound, accuracy is regained and this gain is considerably higher in conditions supported with sound (C_3-AudSupp:PercSoundSupport: *p* = 4.93e-04, C_4-AVRhythmSupp:PercSoundSupport: *p* = 3.676e-02) compared to the unsupported condition C_1-NoSupp. Order has a positive OR indicating an improvement over time but this is counteracted for participants that state they feel tired at the start of the experiment. Perceived task difficulty predicts lower scores while perceived rhythm support predicts an increase in performance on this score.

The Levenshtein distance (L) also assesses performance in a more holistic way but more subtle than the PerDigit score. Condition is included in the final model as a factor and in interaction with PercSoundSupport and MusEducation. Those reporting perceived support by sound score significantly better in C_3-AudSupp compared to C_1-NoSupp (*p* = 1.70e-04) and C_2-VisRhythmSupp (*p* = 7.74e-03) and in C_4-AVRhythmSupp compared to C_1-NoSupp (*p* = 2.056e-02); thus sound seems to increase performance compared to the unsupported condition but not compared to the condition where visual rhythm is already present. However, the model also shows that persons with music education score significantly worse in C_3-AudSupp compared to C_1-NoSupp (*p* = 4.617e-02) and in C_4-AVRhythmSupp compared to C_1-NoSupp (*p* = 5.23e-03) and C_2-VisRhythmSupp (*p* = 4.622e-02) and thus have no benefit of the sound. Significance values can be checked in Table 3. Visual dominance obtained from the pre-test is retained in the model for L with a strong odds ratio showing that persons that are classified as visually dominant score significantly lower in general, but no interaction effect with Dominance was retained in the model and hence this holds for all conditions. Valence indicated on a two-dimensional mood chart (M-Valence) has a low odds ratio showing that high valence results in better performance. As expected, perceived task difficulty has a high odds ratio indicating lower performance. Female gender and Order of the sequence in the test also predict better performance.

## 4. Discussion

In this experiment it was first of all expected to observe an auditory support. On top of this it should be possible to observe a difference between auditory rhythmic and auditory non-rhythmic support. The effect of sound is indeed confirmed, but although supported by multiple well established theories, no rhythmic support was observed. Next to the effect of audio and rhythm also differences due to personal characteristics were found. Quite surprisingly, having received a music education had a negative effect on performance. This section puts the results into perspective and proposes possible explanations.

### 4.1. Difference Between Rhythmic and Non-rhythmic Auditory Support

Statistical analysis of the distribution of the three performance scores revealed that auditory support of the visual target stimuli significantly increases the task performance (i.e., having a more correct sequence recall). On the other hand, rhythmic audio support is not different from non-rhythmic audio support. The models that best represent these distributions are those that do not take into account personal characteristics. Therefore, odds ratios and significance values for models PD1, L1 and D1 are presented in Table 4. These models show a significant positive influence for conditions with auditory support and thus agree with the measured data. Likewise the absence of a rhythmic support effect can be confirmed.

**Table 4:** Odds ratios (OR) of the terms Condition and Order in the X1 models for all three outcome scores.

**Term**	***OR*_*PD*_**	***OR*_*L*_**	***OR*_*D*_**	***p*_*PD*_**	***p*_*L*_**	***p*_*D*_**
C_1-NoSupp	Reference level					
C_2-VisRhythmSupp	0.95159	1.06716	1.10422	0.49284	0.37181	0.16466
C_3-AudSupp	1.33857	0.73039	0.76353	7.56e-05	2.22e-05	1.65e-04
C_4-AVRhythmSupp	1.32516	0.77967	0.86389	1.18e-04	7.10e-04	0.04054
Order	1.00499	0.99519	0.99674	4.01e-11	2.05e-10	8.86e-06

*OR of conditions are in comparison with C_1-NoSupp, while OR of Order is given per unit increase. Next to the OR values, the p-values indicate whether or not the variable significantly contributed to the model*.

When adding personal factors to the models, the auditory conditions C_3-AudSupp and C_4-AVRhythmSupp only showed to be significantly better than the conditions without sound in case of the Distraction score. No matter what values the personal variables take, in these conditions the Distraction score will always be better.

While the benefit of auditory support in a visual task is in line with a body of prior evidence such as the pip-and-pop experiment (Van der Burg et al., 2008), the effect of rhythm is less commonly described. A recent study by Elbaz and Yeshurun (2020) showed that induced rhythm does affect overall alertness but the accuracy of task performance does not depend on the synchronization between the rhythm and the moment of occurrence of the target. This absence of synchronization is similar to our findings. A recent paper by Huygelier et al. (2021) reveals that the role of automatic attention grabbing for the combination of sensory modalities is not straightforward. In their experiments, they found no evidence for the expected attention benefit for synchronous over asynchronous audiovisual looming signals. In addition, also no benefit was found for sustained attention, which was thought to be achieved when a repeated stimulus such as a rhythm is presented. Together these papers emphasize that attentional benefits for multi-sensory signals cannot always be observed for all types of multi-sensory events and thus cannot be automatically expected. The findings could also relate to the discussion whether multi-sensory integration happens at an early, pre-attentive or at a late, attention modulated processing state. Much research has been dedicated to this, but findings remain conflicting. Huygelier et al. (2021) present a detailed discussion about this topic. However, based on the theories of dynamic attending and predictive coding a difference between rhythmic and non-rhythmic support could be expected as they suggest that rhythms generate expectations that open up slots for attending. Visual stimuli occurring at those attended spots would facilitate the memorization and the subsequent recall of the target stimuli. The DAT assumes that the rhythmic entrainment of attention does not depend on the modality (Large and Jones, 1999). Evidence has been found for entrainment within the auditory modality, as well as for a visual rhythm modulating temporal attention to visual targets (Doherty et al., 2005; Correa and Nobre, 2008). Whether the rhythmic entrainment is applicable for cross-modal processing has long been an open issue. In that regard, Escoffier et al. (2010) found that the effect of rhythm on cognition is amodal: attention is entrained such that the processing of information presented in synchrony with the rhythm is facilitated across modalities.

Kahneman (1973) put forward a model where attentional resources are drawn from a general, but limited pool of resources. Depending on other concurrent resource demands, it might be necessary to allocate more or less resources to the specific task. This model might provide a possible explanation why the rhythm does not provide extra support. The main task for the participants is to recall the target sequence and hence memory requires the major part of available resources. As a consequence not enough resources can be assigned to the processing of the rhythmic support. Although this is a possible explanation, the authors argue that precautions were taken in the experimental design to limit the cognitive load. Miller (1956) stated that normal healthy adults have on average a digit span between five and nine. The participants of this experiment were selected to be “normal healthy adults” and the amount of target digits was fixed at five. This number of digits should therefore be rather easy to remember, and thus should not put a big demand on the available cognitive resources.

Also attractor dynamics can possibly provide part of an explanation. Rosso et al. (2021) designed an experiment were a pair of participants were instructed to tap along a metronome. Both metronomes gradually went out of phase from each other. In the visual modality they tapped along with an auditory metronome, while seeing either the partner's tapping hand (visual coupled) or their own tapping hand (visual uncoupled). In the auditory modality, they tapped along with a blinking led, while being presented with the sonification of either the partner's tapping (auditory coupled) or their own (auditory uncoupled). They were explicitly instructed to focus on their own metronome stimulus and neglect the coupled stimulus. While participants in the visual coupled condition were attracted by their partner's tapping and deviated from their own metronome, the auditory rhythm coming from the partner did not attract synchronization when following the visual metronome. This shows similarities with our results, as an auditory rhythm did not attract attention while performing a visual task.

The expected effect of rhythm does not show in the data and the models overall. However, dividing the participants between those that experienced the rhythm as supporting compared to disturbing showed a positive benefit of perceiving rhythmic support. Per unit increase of PercRhythmSupport those people have a 26% lower chance to get a high Levenshtein score / 21% higher chance to achieve a high PerDigit score. The effect was significant in the conditions having rhythm, but was considerable in all four (Figure 3H). In line with the findings of Elbaz and Yeshurun (2020), those that noticed the rhythmic support seemed to have a higher alertness overall and thus performed better. Their attention was mainly directed outwards to better select and integrate the modality-specific sensory input streams coming from the stimuli.

For increasing levels of self-reported PercSoundSupport, the chances of achieving a high PerDigit / Levenshtein score significantly increase / decrease in C_3-AudSupp (PD: *p* = 4.98e-04, L: *p* = 1.70e-04) and C_4-AVRhythmSupp (PD: *p* = 3.672e-02, L: *p* = 2.056e-02), compared to when there is no support. This makes sense as these are the conditions that have sound and is in line with the results in Figure 3E. Participants with higher PercSoundSupport are aware of the support that is provided by the sound and are able to use it in their advantage.

In the model for the Distraction score, the benefit of auditory support decreases with increasing PercTaskDifficulty. In both the PerDigit and Levenshtein model the effect is common for all conditions, not only those with audio. According to Equation 7, participants that experienced the task as very hard compared to very easy (PercTaskDifficulty_5 vs. PercTaskDifficulty_0), have about 75.6% (0.75428^5^ = 0.24415) less chance to achieve a high PerDigit score and about a 378% (1.36736^5^ = 4.77985) higher chance to achieve a high Levenshtein score, and thus performing worse in both cases. When a participant feels like the task was hard to do, they did indeed had a hard time performing well. This finding can confirm the proposed theory where the cognitive load of the task is to high in order to have enough spare resources to incorporate the provided support and benefit from it (Kahneman, 1973).

### 4.2. Personal Effects on Task Performance

Some factors describe the personal characteristics of the participant. A first one is the variable Dominance. When looking at the data (Figure 3D), auditory dominant participants perform better than their visually dominant counterparts in all scores and across all conditions. Although the effect is visible in all three outcome scores, it is only reflected in the model for Levenshtein. Participants that are visually dominant have a 146% (OR 2.46331) higher chance of scoring high in Levenshtein distance, and thus performing worse, compared to those being auditory dominant. It is remarkable that auditory dominance provides a benefit in all four conditions, and not only in those that provide auditory support.

A second factor is MusEducation. It's effect is the biggest in C_4-AVRhythmSupp, while C_2-VisRhythmSupp showed no significant effect. As good performances are reflected in low Levenshtein scores, C_4-AVRhythmSupp leads to the worst performance. This is rather counter intuitive as one would expect to perform better because of the music education, but no evidence that musical training plays a role in this task could be found. However, Figure 3F shows that people with a music education do have a benefit when there is no support at all. But this advantage disappears when sound and/or rhythm are added as extra support. This rather counter-intuitive result can possibly be explained by the earlier proposed theory of limited cognitive load. Musicians might by default use more cognitive resources, which make them perform better by default (C_1-NoSupp). When sound and rhythm are added, not enough cognitive resources are left to optimally integrate them to achieve a better performance. When too much resources need to be assigned to pay attention to the supports and less resources are available to perform the task, the performance even drops (see C_4-AVRhythmSupp in Figure 3F). By calculating the correlation between MusEducation and AVDominance (–0.384), MusEducation and SoundSupport (0.028) and MusEducation and RhythmSupport (0.081) it can be ruled out that an explanation has to be found in the overlapping and interacting effects of these factors. The most personal factor is probably the variable Participant, which is common in all models. This means that depending on the person, scores will by default be different. For instance Participant_30 (OR 6.73146) scores naturally much higher than Participant_11 (OR 0.26086). The variable Participant was treated as a random effect, as there are more possible participants than those that participated in the experiment.

Currently the variables PercSoundSupport and PercRhythmSupport were not treated as variables describing the characteristics of the participant, but rather as variables that describe the state of the participant at a given time. The remark needs to be made that these variables can have an underlying personal variable that was not polled for in the questionnaires or can not be objectively measured. SoundSensitivity (similar to NoiseSensitivity) and RhythmSensitivity could be possibilities in that view. Another variable that describes the state, in this case the mood, of a participant, is the factor M-Valence. It is only significant in the Levenshtein model, were for every unit increase of this variable, the chances of a high score (and thus a worse performance) drop by about 67%. Stated otherwise, performance increases for higher values of M-Valence. An increasing valence corresponds with a more pleasant emotion. All variables that did not end up in the final models did not significantly improve the specific model. The variable Handedness is an exception herein because its interaction with Condition was considerable for all models. However, as Handedness was not balanced for (5 left, 36 right), this would lead to wrong conclusions and the factor is therefore not included. Non-significant variables include MusActive, Age, M-Arousal, Activity, Caffeine, NoiseSensitivity and PercMemoryDifficulty. Their meaning is explained in Section 2.8.

### 4.3. Methodological Issues

While Condition is expected to reflect the results of the intervention with different support conditions, the effects of Sequence and Order are related to the experimental setup. These effects need to be avoided as they can interfere with interpretation of the results. The sequences themselves do have an impact on the result, as some of them are significantly easier (e.g., Sequence24, OR 2.34403) or more complicated (e.g., Sequence15, OR 0.43750) than others (see Figure 4). However, all participants received the same sequences during the experiment and careful pseudo-random design was applied to evenly distribute the individual sequences over the time course of the experiment.

The variable Order mirrors the time in the experiment. The OR of Order is seemingly very small, but at the end of the experiment, the small effect has been applied 119 times and becomes a lot bigger. In all three models performance increases with every unit increase of Order. Overall, this means that participants take a hesitating start and perform better toward the end of the experiment. They gradually get used to the task and learn how to perform better. This phenomenon is therefore called a learning effect. In the opposite case, called a fatigue effect, people do well in the beginning but get fatigued toward the end, causing their performance to drop. To check if the rate of learning is the same across the experiment, a term Order^2^ was added to the multilevel model equations. This term did not significantly improve the models and was removed again. Also interactions with Order were added. A link was expected with Participant as some people would show a fatigue effect and others a learning effect. This is confirmed in the models by means of a significant improvement from X3 to X4 models. For instance, Participant_15 shows a strong learning effect as the odds of getting a high PerDigit score increase by 1.2% (OR 1.01240) for every unit increase of Order. On the other side, the odds of Participant_22 getting a high PerDigit score drop by about 0.84% (OR 0.99162) for every unit increase in Order.

In both the data and the modeling, rhythmic presentation of the targets did not show any extra improvement in performance. This could falsify part of this works hypothesis but some possible methodological issues cannot be excluded. In the rhythmic conditions, five induction stimuli were presented in order to prime the participant with the rhythm. Possibly, this priming was not sustained long enough to have an effect. Moreover, to avoid experimental bias, participants were not made aware of the fact that rhythm could be present during some of the tasks (only one quarter of the sequences had rhythmic auditory support). Because the order of conditions was pseudo-randomized it is not very probable to have several rhythmic sequences next to each other. A previous sequence might create an expectation of rhythmic support in a next one, but chances are small this next one will have rhythm. A block design might avoid this, but would initiate other issues like directing attention toward the support. Besides, in the non auditory rhythmic condition (C_2-VisRhythmSupp) the rhythm was induced visually by means of flashing empty circles. It is known that synchronizing to a periodically flashing visual stimulus (a static stimulus) is difficult and can be substantially improved with a moving stimulus like a bouncing ball (Hove et al., 2013). The combination of these issues could be a reason why the rhythm was not induced strong enough to update predictions about the sensory inputs. As stated previously and based on the directed attention hypothesis, no special mention was made about the audio or rhythm in order not to draw attention to it. As a consequence there might have been more conscious attention to the rhythmically presented stimuli in some subjects and/or in some parts of experimental sessions. This might in turn have influenced the performance, as it has been claimed that conscious attention to both sensory modalities simultaneously is essential for enhanced performance (van Ee et al., 2009). This could explain part of the variability in the data. However, based on the knowledge from pilot data, instructing the participants to pay attention to the rhythm would have made the task too easy and would therefore require major changes to the experimental design.

Last but not least, a remark needs to be made about the used scores. The PerDigit and Levenshtein scores are closely related to working memory. The use of working memory to address attention can be justified based on the literature, as these two processes are inherently linked to each other (Cowan, 2001; Kiyonaga and Egner, 2013; Sörqvist and Rönnberg, 2014; Vetter et al., 2014; Quak et al., 2015; Talsma, 2015). The authors believe that the developed Distraction score is more directly linked with attention. If attention was not involved, participants would equally make mistakes in both the target and distractor stimuli. Last but not least, the attentional component in the processing of stimuli is composed of both auditory and visual attention. These two components leave their mark in different ways, and thus some aspects of them should definitely have an effect on the performance.

### 4.4. Future Work

Our study had a focus on the behavioral outcome of a task involving visual attention, memory and sequence recall. In order to better understand the role of attention, in future work we need to consider the direct measurement of brain activation, for instance via electroencephalography (EEG). For instance the amplitude of the P300 has been shown to be proportional to the amount of attentional resources that are available for stimulus processing (Johnson, 1988; Gray et al., 2004). This link could give insights into the hypothesized clash between cognitive and attentional resources. Besides, it allows to hypothesize that there is a variation in gating and attention capabilities at the neurological level. This variation, together with other variables derived from the EEG results, can then be added to the personal aspects in the models to further explain away the person dependence.

## 5. Conclusion

This work aimed to contribute to a better understanding of multi-sensory processing, attention and working memory. A novel paradigm was developed that used a rapid stream of visual target digits and distractor digits. Depending on the condition, either no support was given in synchrony with the target stimuli, or they were supported by rhythm, by sound or by the combination of sound and rhythm. Participants had to memorize and recall the sequence of target digits. Firstly, it was hypothesized that the memory recall is improved by auditory support, which is clearly confirmed by our results. Based on the dynamical attending and predictive coding theories, we expected to see a global difference between rhythmic and non-rhythmic sounds. This could not be seen in the measured data and could not been simulated by means of our models. However, individual differences were observed. Participants that indicated they perceived the rhythm as supportive did show improved performance in rhythmic conditions. A rather surprising effect was observed for people with a music education. Counter intuitively, they performed worse than their non-trained counterparts.

## Data Availability Statement

The datasets presented in this study can be found in online repositories. The names of the repository/repositories and accession number(s) can be found below: https://osf.io/ntmy8/?view_only=88d951c394c7481dba00a1497d64797f.

## Ethics Statement

The studies involving human participants were reviewed and approved by the Ethics Committee of the faculty of Arts and Philosophy, Ghent University. The participants provided their written informed consent to participate in this study.

## Author Contributions

JDW prepared and carried out the measurements and wrote the manuscript and designed the figures with support and input from PD, ML, and DB. PD, ML, and DB supervised the project and provided critical feedback in all stages of the study. All authors discussed the results, contributed to its interpretation, designed the study and planned the experiments.

## Funding

This research was funded by the Research Foundation-Flanders under Grant No. G0A0220N.

## Conflict of Interest

The authors declare that the research was conducted in the absence of any commercial or financial relationships that could be construed as a potential conflict of interest.

## Publisher's Note

All claims expressed in this article are solely those of the authors and do not necessarily represent those of their affiliated organizations, or those of the publisher, the editors and the reviewers. Any product that may be evaluated in this article, or claim that may be made by its manufacturer, is not guaranteed or endorsed by the publisher.
